# Biological Characteristics and Clinical Significance of Soluble PD-1/PD-L1 and Exosomal PD-L1 in Cancer

**DOI:** 10.3389/fimmu.2022.827921

**Published:** 2022-03-21

**Authors:** Mengke Niu, Yiming Liu, Ming Yi, Dechao Jiao, Kongming Wu

**Affiliations:** ^1^ Department of Oncology, Tongji Hospital of Tongji Medical College, Huazhong University of Science and Technology, Wuhan, China; ^2^ Department of Interventional Radiology, The First Affiliated Hospital of Zhengzhou University, Zhengzhou, China

**Keywords:** soluble PD-1, soluble PD-L1, cancer, biological activity, efficacy prediction, prognosis, immunotherapy, exosomal PD-L1

## Abstract

The immune checkpoint pathway consisting of the cell membrane-bound molecule programmed death protein 1 (PD-1) and its ligand PD-L1 has been found to mediate negative regulatory signals that effectively inhibit T-cell proliferation and function and impair antitumor immune responses. Considerable evidence suggests that the PD-1/PD-L1 pathway is responsible for tumor immune tolerance and immune escape. Blockage of this pathway has been found to reverse T lymphocyte depletion and restore antitumor immunity. Antagonists targeting this pathway have shown significant clinical activity in specific cancer types. Although originally identified as membrane-type molecules, several other forms of PD-1/PD-L1 have been detected in the blood of cancer patients, including soluble PD-1/PD-L1 (sPD-1/sPD-L1) and exosomal PD-L1 (exoPD-L1), increasing the composition and functional complications of the PD-1/PD-L1 signaling pathway. For example, sPD-1 has been shown to block the PD-1/PD-L immunosuppressive pathway by binding to PD-L1 and PD-L2, whereas the role of sPD-L1 and its mechanism of action in cancer remain unclear. In addition, many studies have investigated the roles of exoPD-L1 in immunosuppression, as a biomarker for tumor progression and as a predictive biomarker for response to immunotherapy. This review describes the molecular mechanisms underlying the generation of sPD-1/sPD-L1 and exoPD-L1, along with their biological activities and methods of detection. In addition, this review discusses the clinical importance of sPD-1/sPD-L1 and exoPD-L1 in cancer, including their predictive and prognostic roles and the effects of treatments that target these molecules.

## 1 Introduction

The immune cycle consists of a series of reduplicative events required by the immune system to generate a robust antitumor immune response (1). Neoantigens on tumors are recognized by antigen-presenting cells (APCs), which activate tumor antigen-specific T lymphocytes to eliminate cancer cells (1). Antitumor immunity is also mediated by several negative factors, such as programmed death protein 1 (PD-1), programmed death ligand-1 (PD-L1), and cytotoxic T-lymphocyte-associated antigen-4 (CTLA-4) (2, 3). The binding of the immunosuppressive molecule PD-1 to its ligand PD-L1 can initiate programmed T cell death, induce the production of Foxp3^+^CD4^+^ regulatory T cells (Tregs) and enhance the function of immunosuppressive Tregs (4, 5). The immunosuppressive microenvironment provided by negative regulatory signaling pathways is an important component of tumor escape from the immune system in cancer patients (6).

Although mainly expressed on T cells, PD-1 is also presented on other immune cells including natural killer (NK) cells, monocytes, dendritic cells (DCs), B cells, and Tregs (7). PD-1 expression is induced by multiple cytokines, such as interferon-α (IFN-α), released by DCs and monocytes (8). PD-L1 is mainly expressed on the surface of tumor cells, but is also presented on immune system cells and non-hematopoietic cells, including vascular endothelial and epithelial cells (7). Oncogenic signals, such as PI3K-AKT, MEK-ERK, and EGFR (9–11), as well as several cytokines, such as interleukin-6 (IL-6) and IFN-γ (12, 13), have been found to regulate PD-L1 expression. The binding of PD-L1 to PD-1 triggers PD-1-mediated intracellular signaling and inhibits the PI3K-AKT and MAPK pathways, thereby restricting T-cell proliferation, activation and survival (14, 15). In addition, the complete activation of T cells relies on two signals, with the first signal provided by the specific binding of T-cell receptor (TCR) to major histocompatibility complex (MHC) and the second signal provided by the interaction of CD28 on T cell surfaces with co-stimulatory molecules expressed by APCs (16). The mechanism of PD-1/PD-L1 signaling involves the recruitment of the Src homology 2 domain containing phosphatases 2 (SHP2) to the PD-1 cytoplasmic domain, which dephosphorylates signaling molecules of the proximal TCR and CD28 pathways (17). PD-L1 can also mediate reverse signaling that upregulates the PI3K-AKT signaling cascade and enhances glycolytic metabolism in cancer cells (18).

Many studies have indicated the potential importance of membrane-bound PD-1/PD-L1 (mPD-1/mPD-L1) in antitumor immunotherapy (19, 20). Blocking the interaction of PD-1 with PD-L1 improves T-cell function, restarting and amplifying the cancer-immune cycle (1). Hence, anti-PD-1/PD-L1 antibodies are frequently used to treat various types of solid tumors and have greatly improved the survival of patients with advanced cancers (21, 22). Clinical responses to antitumor treatment strategies, including immunotherapy, have been found to vary widely across different cancer types, and the durability of these responses varies from patient to patient (23–25). This may be due in part to the form of PD-1/PD-L1 presentation (26). In recent years, soluble PD-1/PD-L1 (sPD-1/sPD-L1) and exosomal PD-L1 (exoPD-L1) have been detected in the blood of tumor patients (17, 27), with many studies investigating the exact roles of these soluble molecules in cancer (28, 29). This review discusses the origin, biological mechanisms and methods of detection of sPD-1/sPD-L1 and exoPD-L1 and highlights their predictive and prognostic roles in cancer, as well as the effects of treatments that target these molecules.

## 2 Generation of sPD-1 and sPD-L1

A variety of immunomodulatory molecules exist as both cell membrane and soluble forms. Many soluble co-stimulatory and co-inhibitory molecules, such as sCTLA-4, sCD80, sCD86, sB7-H3, and sLAG-3, have been detected in the blood of cancer patients (30–35). These soluble molecules are produced either by selective splicing of genes encoding the membrane-bound molecules or by proteolytic cleavage of membrane-bound proteins (36, 37). Soluble forms of PD-1 and PD-L1 (sPD-1/sPD-L1) have also been detected in the blood of cancer patients.

### 2.1 Generation of sPD-1

**Figure 1:**
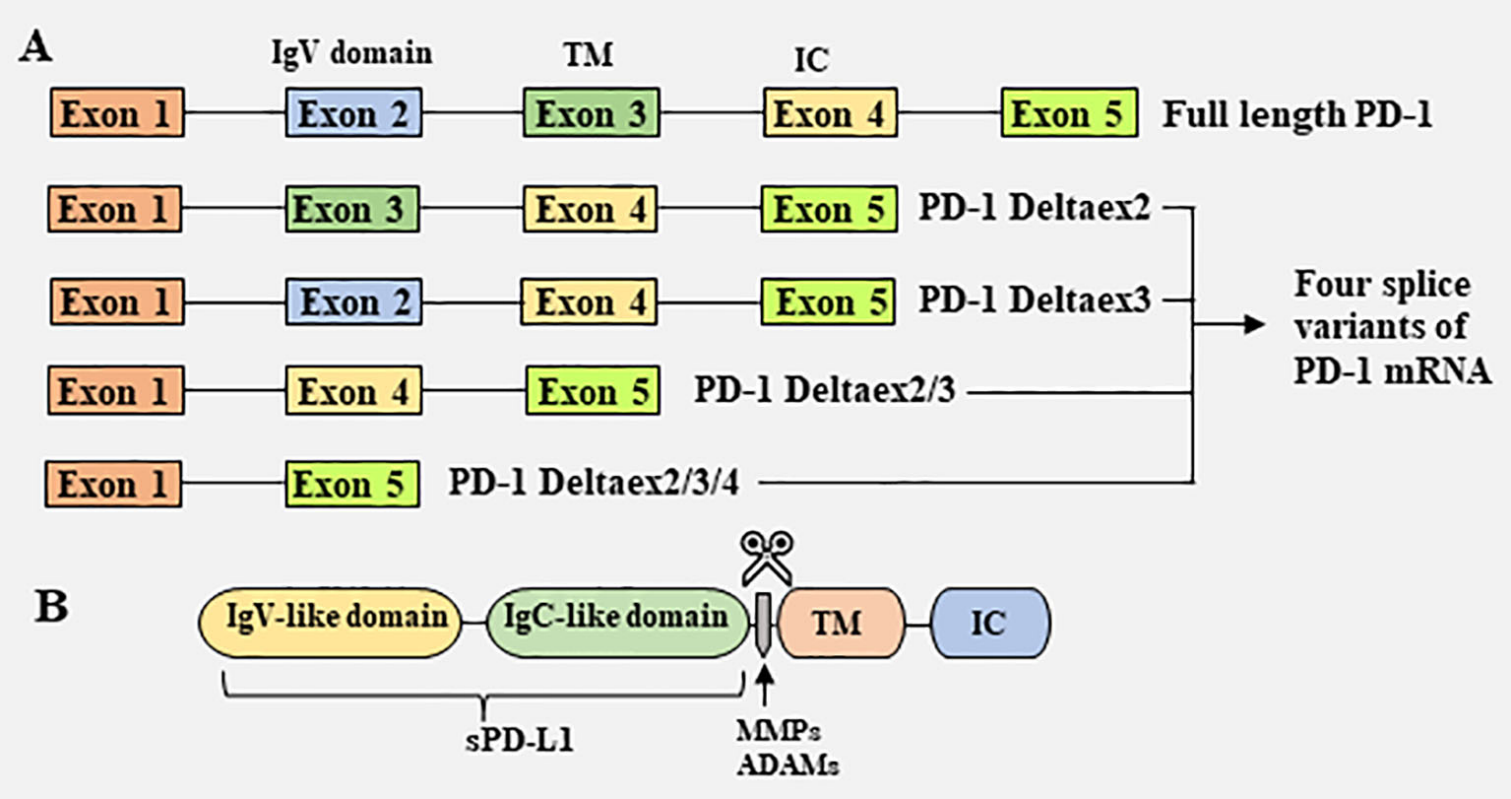
Mechanisms responsible for the generation of soluble PD-1 and PD-L1. **(A)** Generation of soluble PD-1 from splice variants of PD-1 mRNA. Full length PD-1 (flPD-1) mRNA contains five exons (exons 1–5), which encode a short signal sequence, an immunoglobulin (Ig) domain, the stalk and transmembrane (TM) domain, a 12 amino acid **(aa)** sequence that marks the beginning of the cytoplasmic domain, the C-terminal intracellular (IC) domain and a long 3’UTR, respectively. Four splice variants of PD-1 mRNA, PD-1Deltaex2, PD-1Deltaex3, PD-1Deltaex2/3, and PD-1Deltaex2/3/4, have been cloned from human peripheral blood mononuclear cells (PBMCs). PD-1Deltaex3 only lacks exon 3, but retains an extracellular Ig domain. Translation of this mRNA results in a soluble form PD-1. **(B)** Generation of soluble PD-L1 by proteolytic cleavage of membrane-bound PD-L1. Several proteases, including MMPs and ADAMs, are capable of cleaving membrane-bound PD-L1, releasing soluble PD-L1.

sPD-1 is thought to be generated primarily by selective splicing (**Figure 1**) (38). PD-1 is a type I transmembrane glycoprotein encoded by the *Pdcd1* gene on chromosome 2 in humans and chromosome 1 in mice (39). The *Pdcd1* gene contains five exons (exons 1-5), which encode a short signal sequence, an immunoglobulin (Ig) domain, the stalk and transmembrane domain, a short 12 amino acid (aa) sequence that marks the beginning of the cytoplasmic domain, the C-terminal intracellular residues and a long 3’UTR, respectively (39). In addition to full length PD-1 (flPD-1), four splice variants of PD-1 mRNA (PD-1Deltaex2, PD-1Deltaex3, PD-1Deltaex2/3, and PD-1Deltaex2/3/4) have been cloned from human peripheral blood mononuclear cells (PBMCs) (38). PD-1Deltaex2, which lacks exon 2, encoding the extracellular domain; PD-1Deltaex2/3, which lacks exons 2 and 3, encoding the extracellular and transmembrane domains; and PD-1Deltaex2/3/4, which lacks exons 2, 3 and exon 4, and contains a premature stop codon in exon 5, are unable to bind to the ligand PD-L1 and transmit inhibitory signals. In contrast, PD-1Deltaex3, which lacks only exon 3, retains the extracellular Ig domain and can be translated to generate sPD-1 (38).

### 2.2 Generation of sPD-L1

sPD-L1 has been reported to be generated in and released from tumor cells and mature DCs, but not from immature DCs, T cells, macrophages, and monocytes (40). Although both myeloid cells and activated T lymphocytes exhibit elevated levels of mPD-L1, only myeloid cells can produce sPD-L1 (40), suggesting differences in the mechanisms that regulate mPD-L1 and sPD-L1 production. High levels of sPD-L1 have been detected in the supernatants of multiple PD-L1^+^ cell lines (41). Many studies have shown that sPD-L1 is generated by proteolytic cleavage of mPD-L1 by endogenous matrix metalloproteinases (MMPs) (**Figure 1**) (42, 43). PD-L1/PD-L2 expressed on fibroblasts can be cleaved by MMP-9 and MMP-13, resulting in the lack of a PD-1 binding domain, thereby reversing T-cell depletion and limiting immunosuppressive capacity (42). MMP-7 and MMP-13, which are upregulated in the human head and neck squamous cell carcinoma OSC-20 cell line, can cleave PD-L1, whereas inhibitors that specifically target MMP-13 are able to restore mPD-L1 expression (43). MMP inhibitors have also been found to suppress sPD-L1 secretion into the supernatants of PD-L1 transfected cell lines (41). Members of a disintegrin and metalloproteinase (ADAM) family are also involved in the shedding of extracellular domains of membrane molecules (26). For example, ADAM10 and ADAM17 have been found to cleave mPD-L1 on cancer cells and extracellular vesicles (44–46). Other enzymes may also participate in the hydrolytic shedding of membrane molecules and the release of sPD-L1.

PD-L1 is a type I transmembrane glycoprotein encoded by the *CD274* gene on chromosome 9 in humans (47). Transcription of this gene can produce multiple PD-L1 splice variants, including PD-L1 lncRNA splice isoforms and truncated PD-L1 (48, 49). sPD-L1 has been shown to be produced primarily through the exaptation of an intronic *LINE-2A (L2A)* endogenous retroelement in the *CD274* gene, with the resulting CD274-L2A encoded sPD-L1 having a receptor antagonistic effect (50). In addition, exon 4-enriched variants are able to produce a secreted form of PD-L1 in various cancers (49). Secreted PD-L1 binds PD-1 and negatively regulates T-cell function (49). In contrast, one splice variant does not include splicing of the transmembrane domain but secretes PD-L1 with a unique 18 aa tail. This variant is able to homodimerize and more effectively inhibit lymphocyte function (51). A variant lacking exon 2, which encodes the IgV domain, was found in human PBMCs; this was unable to bind PD-1 and to generate sPD-L1 (52).

## 3 Biological Activity

Immunosuppression induced by mPD-1/mPD-L1 was found to be involved in tumor escape from immune system (6). In addition to being presented at the plasma membrane, PD-1/PD-L1 has been detected in the plasma and serum of patients with several diseases (53–56). The biological activity of sPD-1/sPD-L1 is similar to that of the corresponding membrane-bound molecules. Specifically, sPD-1/sPD-L1 may transfer signals among different types of immune cells, altering their activity and regulating the secretion of cytokines (57, 58).

### 3.1 Biological Activity of sPD-1

**Figure 2:**
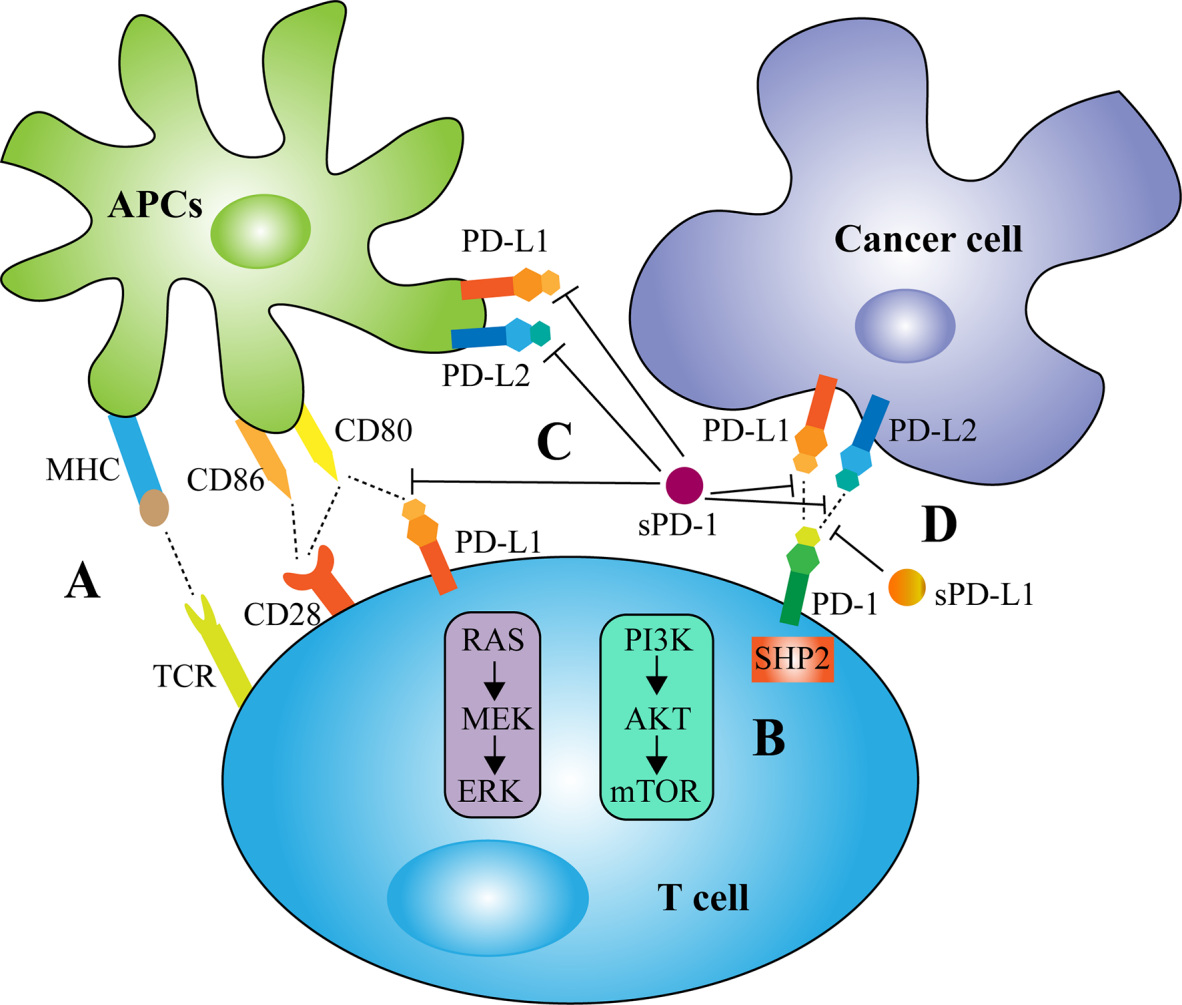
Biological activity of membrane and soluble PD-1/PD-L1 in tumor immunity. **(A)** The complete activation of T cells relies on two signals, with the first signal provided by the specific binding of T-cell receptor (TCR) to major histocompatibility complex (MHC) and the second signal provided by the interaction of CD28 on T cell surfaces with co-stimulatory molecules CD80/CD86 expressed by antigen-presenting cells (APCs). **(B)** The mechanism of PD-1/PD-L1 signaling involves the recruitment of the Src homology 2 domain containing phosphatases 2 (SHP2) to the PD-1 cytoplasmic domain, which dephosphorylates signaling molecules of the PI3K-AKT and MAPK pathways, thereby restricting T-cell proliferation, activation and survival. **(C)** sPD-1 has been found to suppress the interactions of PD-1 with PD-L1 and PD-L2 and the interaction of PD-L1 with CD80. However, sPD-1 may also have reverse signaling effects on APCs *via* the PD-L1/PD-L2 pathway and inhibits T cell function. **(D)** sPD-L1, like mPD-L1, binds to PD-1 to transmit negative regulatory signals.

sPD-1 has been found to function as a blocker of PD-1 ligands and to suppress the interactions of PD-1 with PD-L1 and PD-L2 and the interaction of PD-L1 with B7-1 (CD80) (**Figure 2**) (57). Coadministration of the PD-1/PD-L1 blocker sPD-1 and human papillomavirus-16 E7 DNA vaccine significantly enhanced antitumor activity against E7-expressing tumors (57). Mechanistically, sPD-1 effectively increased the numbers and functional activities of E7-specific CD8^+^ T cells and the maturation of DCs through the upregulation of MHC class II molecules (57). Similarly, reconstructed adeno-associated virus-mediated delivery of the PD-1 extracellular domain to tumor sites augmented the cytotoxicity of antigen-specific lymphocytes (59). In addition, the recombinant peptide sPD-1-CH50 was able to upregulate the expression of IFN-γ, tumor necrosis factor-α (TNF-α), and inducible nitric oxide synthase (iNOS) and to enhance the lytic activity of macrophages against B7-H1-positive tumor cells (60). The combination of heat shock protein 70 (HSP70) and sPD-1 vaccine increased the expression of the TH1 cytokines IL-2 and IFN-γ and decreased the expression of the negative regulatory molecules Foxp3, IL-10 and TGF-β in tumor-infiltrating lymphocytes of lung metastatic melanoma (61). Residual tumor cells expressing B7-H1 were shown to be responsible for tumor resistance to HSP70 vaccine. Intravenous administration of a plasmid encoding the extracellular domain of sPD-1 was able to block the PD-1 pathway, overcome tumor resistance and reduce lung metastasis of B16F1 melanoma cells (61). A eukaryotic expression plasmid expressing sPD-1 was found to partially enhance the early activation of lymphocytes *in vitro*, upregulating the levels of TNF-α, IFN-γ, B7.1, and 4-1BB mRNAs and downregulating the levels of OX40 and IL-10 mRNAs (62). Conversely, sPD-1 may also have reverse signaling effects on DCs *via* the PD-L1/PD-L2 pathway, reducing DC maturation and inhibiting T cell activation and IL-2 production (58, 63).

### 3.2 Biological Activity of sPD-L1

The biological activity of sPD-L1 remains unclear. In most cases, sPD-L1, like mPD-L1, binds to PD-1 to transmit negative regulatory signals (**Figure 2**) (49). A variety of malignant cells and DC-derived sPD-L1 have been reported to induce apoptosis of T cells and impair their function (40, 49, 64, 65). sPD-L1 in the sera of patients with cystic echinococcosis was found to significantly reduce the expression of TH1 cytokines such as IL-2 and to increase the expression of TH2 cytokines such as IL-4, IL-6, and IL-10 (66). Similarly, sPD-L1 in the supernatants of PD-L1-expressing breast cancer cell lines was found to have a negative regulatory effect on cellular immunity (67). The dimerized form of sPD-L1 was found to possess superior immunosuppressive activity compared with monomeric sPD-L1 (51). However, the impact of sPD-L1 on immune cell function may also depend on the affinity between sPD-L1 and PD-1 (68). Native soluble human PD-L1 (hPD-L1) was found to exert a suppressive effect on activated T cells (68). The soluble hPD-L1 variants L3C7-hPD-L1 and L3B3-hPD-L1, which were generated by directed molecular evolution and had a >20-fold higher affinity to PD-1 than native hPD-L1, were able to attenuate the inhibitory effects of the PD-1 axis on PBMC amplification and IFN-γ release (68). A similar result was observed in patients with diabetic atherosclerotic macrovascular diseases (69). These findings suggest that sPD-L1 may also act as a PD-1 blocker, competitively suppressing the inhibitory effect of mPD-L1. In addition, sPD-L1 levels in maternal blood were found to increase throughout gestation and return to control levels post-partum (70). During placental development, IFN-β secretion by decidual macrophages was shown to enhance the constitutive production of sPD-L1 by trophoblasts, initiating macrophage polarization towards an M2 phenotype and thereby reducing inflammation (71). This may at least partly suppress maternal immunity and play a role in immune tolerance that is critical for the fetus (72).

## 4 Detection of sPD-1 and sPD-L1

sPD-1 and sPD-L1 circulate in the blood as free proteins and can be measured by enzyme-linked immunosorbent assays (ELISA) (70, 72, 73). ELISA, however, involves the use of monoclonal antibodies, which are costly to produce and time-consuming to isolate and purify (74). In addition, these assays are complicated to perform and have limited reproducibility and sensitivity for clinical applications. Several new, inexpensive, accurate, sensitive and rapid detection methods have been developed to overcome these limitations.

In a fully automated immunoassay system based on chemiluminescent magnetic technology (HISCL system), specific biotinylated antibodies bind to the target proteins, with the resulting complexes mixed homogeneously with alkaline phosphatase (ALP)-coupled antibodies (75). The fluorescent signals are subsequently measured and the chemiluminescent intensity determined (75). This system is rapid, sensitive and reproducible, and can accurately measure sPD-1, sPD-L1 and sCTLA-4 levels in the clinic (75). Another assay uses localized surface plasmon resonance (LSPR) biosensors (74). Gold nanoshells have strong optical properties of absorption and scattering, with changes in the sizes of these gold nanoshells altering the LSPR absorption peaks. These biosensors can be fabricated using excessively tilted fiber gratings (ExTFGs) coupled with large-sized gold nanoshells (∼160 nm) so that it works at the C/L band (1525 nm-1625 nm) (74). Anti-sPD-L1 monoclonal antibodies are bound to the surface of the ExTFG-LSPR sensor using the staphylococcal protein A (SPA) method, resulting in the label-free and specific detection of sPD-L1 (74). The interaction of sPD-L1 with the corresponding monoclonal antibody has also been shown to enhance the LSPR effect and improve specificity (74).

Another assay system involves the use of an integrated surface enhanced Raman scattering (SERS) microfluidics device with high specificity for the detection of multiple immune checkpoints (76, 77). These biosensors offer several advantages. First, the replacement of monoclonal antibodies with engineered high-affinity nano yeast single chain variable fragments (scFv) results in high efficiency and stability, along with lower cost. In addition, the graphene oxide functionalized surface reverses the general biotin-streptavidin chemistry paradigm; the microfluidics platform induces nanofluidic mixing by ac-electrohydrodynamics (ac-EHD) and integrates microfluidic sandwich immunoassays, which enhance scFv-target specific binding and minimize non-specific binding (76, 77). This biosensor is capable of analyzing up to 28 samples simultaneously in <2 hours and requires only 20 µl of sample per target immune checkpoint (77).

## 5 Clinical Importance of sPD-1 and sPD-L1 in Cancer

### 5.1 Predictive and Prognostic Significance

The levels of sPD-1 and sPD-L1 in cancer patients have been reported to correlate with disease severity, clinicopathological characteristics, survival and response to treatment. Because of their clinical importance, sPD-1 and sPD-L1 have been widely utilized as biomarkers to predict treatment efficacy and patient prognosis (**Tables 1**, **2**).

**Table 1:** Soluble PD-1 expression levels in different cancers and their correlation with disease prognosis and efficacy prediction.

Cancer type	Patients number	Treatment	Principal findings	Reference
NSCLC	38	Erlotinib	34% of patients showed an increase in sPD-1 during erlotinib treatment; Increased sPD-1 during treatment was associated with prolonged PFS (adjusted HR 0.32, *p* = 0.013) and OS (adjusted HR 0.33, *p* = 0.006).	(78)
NPC	77	IMRT	IMRT could increase the expression of sPD-1; The expression level of sPD-1 in TNM I/II patients was significantly higher than that in III/IV patients; Patients with high sPD-1 had longer survival than those with low sPD-1.	(79)
NSCLC	87	Nivolumab	After two cycles of nivolumab, an increased or stable sPD-1 level independently correlated with longer PFS (HR 0.49, 95%CI (0.30-0.80), *p* = 0.004) and OS (HR 0.39, 95%CI (0.21-0.71), *p* = 0.002).	(28)
HCC	120	Radical resection	sPD-1 was a favorable independent prognostic factor (DFS, HR 0.32, 95%CI (0.14-0.74), *p* = 0.007; OS, HR 0.54, 95%CI (0.30-0.98), *p* = 0.044).	(53)
Advanced rectal cancer	117	CRT	High sPD-1 before and after CRT was significantly associated with longer distance of the tumor from the anal verge.	(80)
PDAC	32	/	Plasma level threshold that correlates with less than six months survival was established for sPD-1 (>8.6 ng/ml).	(81)
DLBCL	121	Immunochemotherapy	The relative risk of death was 2.9-fold (95%CI (1.12-7.75), *p* = 0.028) and the risk of progression was 2.8-fold (95%CI (1.16-6.56), *p* = 0.021) in patients with high pretreatment sPD-1 levels	(82)

PD-1, soluble programmed death protein 1; NSCLC, non-small cell lung cancer; PFS, progression-free survival; HR, hazard ratio; OS, overall survival; NPC, nasopharyngeal carcinoma; IMRT, intensity-modulated radiation therapy; CI, confidence interval; HCC, hepatocellular carcinoma; DFS, disease-free survival; CRT, chemoradiotherapy; PDAC, pancreatic ductal adenocarcinoma; DLBCL, diffuse large B-cell lymphoma.

**Table 2:** Soluble PD-L1 expression levels in different cancers and their correlation with disease prognosis and efficacy prediction.

Cancer type	Patients number	Treatment	Principal findings	Reference
NSCLC	233	Pembrolizumab or nivolumab	The disease control rate in the high sPD-L1 group was significantly lower than that in the low sPD-L1 group (37% vs. 57%, *p* = 0.0158); The high levels of serum sPD-L1 were independently associated with shorter PFS (HR 1.910; *p* = 0.061) and OS (HR 2.073; *p* = 0.034).	(83)
ESCC	153	/	sPD-L1 levels in patients with high PD-L1 expression levels in tumor tissue were significantly higher (*p* = 0.042); The OS of the sPD-L1-high group was significantly worse (*p* = 0.028).	(84)
OC	53	/	OC patients with a higher level of sPD-L1 in the peritoneal fluid had shorter 5-year survival than those with a lower sPD-L1 concentration (median 48 vs. 27 months).	(85)
Advanced rectal cancer	117	CRT	sPD-L1 levels were significantly increased after CRT. High sPD-L1 after CRT was associated with lymphovascular invasion and poorer DFS.	(80)
ESCC	190	Cytotoxic chemotherapy	Median OS of sPD-L1-high patients was lower than in patients with low sPD-L1 level (12 vs. 21 months, *p* < 0.001).	(86)
ccRCC	89	/	sPD-L1 was higher for metastatic patients compared to non-metastatic patients.	(87)
BC	132	/	Significantly higher serum sPD-L1 levels were found in patients with muscle invasive disease and metastatic disease (*p* < 0.05).	(88)
Urothelial Cancer	95	Chemotherapy or ICIs	High baseline sPD-L1 levels were associated with worse ECOG status (*p* = 0.007) and shorter OS for both chemotherapy- and ICI-treated patients (*p* = 0.002 and 0.040, respectively).	(89)
Glioma	60	RT	The baseline sPD-L1 levels were significantly associated with tumor grade, IDH-1 mutation status and Ki-67 expression; PFS and OS were significantly worse in patients with higher baseline levels of sPD-L1 (*p* = 0.027 and 0.008, respectively).	(90)
HCC	121	/	Patients with high sPD-L1 value (>96 pg/ml) had worse DFS and OS (HR 5.42, 95%CI (2.28-12.91), *p* < 0.001, and HR 9.67, 95%CI (4.33-21.59), *p* < 0.001).	(91)
STS	135	/	The high sPD-L1 (>44.26 pg/ml) group had significantly lower MS and lower OS than the low sPD-L1 group (≤44.26 pg/ml) at 5 years (42.4% vs. 88.4%, *p* < 0.001, and 64.1% vs. 89.2%, *p* = 0.011).	(92)
Melanoma	100	ICIs	Pretreatment levels of sPD-L1 were elevated in stage IV melanoma patient sera compared with healthy donors; High pretreatment levels of sPD-L1 were associated with increased likelihood of progressive disease in patients treated by ICIs; Elevated sPD-L1 after checkpoint blocker treatment was associated with PR.	(65)
Lung cancer	1188	ICIs	High sPD-L1 predicted worse OS (HR 1.60, 95%CI (1.31-1.96), *p* < 0.001) and lower ORR (odds ratio 0.52, 95%CI (0.35-0.80), *p* = 0.002) in patients treated with non-ICI therapies. High sPD-L1 was significantly associated with worse OS (HR 2.20, 95%CI (1.59-3.05), *p* < 0.001) and PFS (HR 2.42, 95%CI (1.72-3.42), *p* < 0.001) in patients treated with ICIs.	(93)

PD-L1, programmed death ligand 1; NSCLC, non-small cell lung cancer; PFS, progression-free survival; HR, hazard ratio; OS, overall survival; ESCC, esophageal squamous cell carcinoma; OC, ovarian cancer; CRT, chemoradiotherapy; DFS, disease-free survival; ccRCC, clear cell renal cell carcinoma; BC, bladder cancer; ICIs, immune checkpoint inhibitors; RT, radiotherapy; IDH-1, isocitrate dehydrogenase-1; HCC, hepatocellular carcinoma; CI, confidence interval; STS, soft tissue sarcoma; MS, metastasis-free survival; PR, partial response; ORR, objective response rate.

#### 5.1.1 Predictive and Prognostic Significance of sPD-1

Erlotinib is a tyrosine kinase inhibitor that inhibits the activity of the epidermal growth factor receptor (EGFR) (94). In a study of patients with non-small cell lung cancer (NSCLC), 34% of erlotinib-treated patients showed elevated sPD-1 during treatment, with these patients experiencing prolonged progression-free survival (PFS) and overall survival (OS) compared with patients without elevated sPD-1 (78). Similarly, sPD-1 was significantly elevated in patients with nasopharyngeal carcinoma after intensity-modulated radiation therapy (IMRT), with survival being longer in patients with high than with low sPD-1 (79). In addition, sPD-1 expression was significantly higher in TNM I/II patients than in TNM III/IV patients (79). Radiotherapy may enhance the antigen-presentation process, which increases the number of tumor-specific T lymphocytes and sPD-1 production (95, 96). NSCLC patients with increased or stable sPD-1 after anti-PD-1 therapy also tended to have favorable outcomes (28). In addition, a retrospective study of patients with hepatocellular carcinoma (HCC) undergoing radical resection reported that enhanced sPD-1 was associated with longer survival (53). The mechanisms by which sPD-1 is increased and patient survival improved after specific treatments, such as targeted therapy, radiotherapy, and immunotherapy, are not fully understood. These treatments may affect the cancer-immune cycle, such as by increasing antigen presentation, restoring tumor-specific cytotoxic T-cell activity and enhancing anti-cancer immunity (95–98). In contrast, elevated sPD-1 in untreated cancer patients was found to predict unfavorable survival outcomes. High sPD-1 in patients with advanced rectal cancer correlated with a longer distance of the tumor from the anal verge (80). In addition, plasma sPD-1 concentration >8.6 ng/ml was found to be associated with OS < 6 months in 27 patients newly diagnosed with pancreatic ductal adenocarcinoma (81). Pretreatment sPD-1 levels are a predictor of adverse outcomes after dose-dense immunochemotherapy and can predict the risk of disease progression in patients with diffuse large B-cell lymphoma (DLBCL) (82). Additional studies are required determine the precise mechanism underlying the relationship between malignancy and sPD-1.

#### 5.1.2 Predictive and Prognostic Significance of sPD-L1

In general, elevated sPD-L1 in cancer patients is indicative of poor prognosis or resistance to treatment (83–85). High sPD-L1 levels before neoadjuvant chemoradiotherapy (CRT) were found to be associated with younger age, and significantly increased sPD-L1 levels after CRT were associated with lymphovascular infiltration and shorter disease-free survival (DFS) (80). High pretreatment sPD-L1 concentration may be a marker of poor prognosis in patients with locally advanced or metastatic esophageal squamous cell carcinoma treated with cytotoxic chemotherapy (86). Higher sPD-L1 levels were observed in patients with metastatic than non-metastatic clear cell renal cell carcinoma (87), and sPD-L1 levels were significantly elevated in patients with muscle-invasive and metastatic urinary bladder cancer (88). Similarly, high baseline sPD-L1 levels were associated with poorer ECOG status and shorter OS in patients treated with chemotherapy and immune checkpoint inhibitors (ICIs) (89). Higher baseline sPD-L1 levels in patients with glioma treated with radiotherapy were associated with poorer PFS and OS (90), and high sPD-L1 was considered a biomarker of poor prognosis in HCC patients undergoing curative treatment (91). High sPD-L1 concentrations were also found to predict future metastases in patients with soft tissue sarcoma (92), and high pretreatment sPD-L1 was associated with an increased likelihood of disease progression in patients with malignant melanoma (65). In addition, high sPD-L1 was significantly associated with poorer OS and PFS in lung cancer patients treated with ICIs (93).

### 5.2 Therapeutic Value

In recent years, immune checkpoint blockade has attracted considerable attention in cancer immunotherapy, with anti-PD-1/PD-L1 treatments associated with high response rates in multiple advanced-stage cancers (99–103). sPD-1 and sPD-L1 were found to have immunomodulatory activities, suggesting that agents that modify their activities may have application in cancer treatment. These agents have been tested in animal models, with the results of these studies possibly providing new insights for future anti-cancer therapy in humans.

#### 5.2.1 Therapeutic Value of sPD-1

sPD-1 has been found to enhance the effector functions of T lymphocytes and other immune cells (57). Constitutive sPD-1-secreting sPD-1 chimeric antigen receptor (CAR) T cells were found to mitigate tumor burden and prolong OS in NOD-SCID-IL2rg mice bearing NALM-6-PD-L1 (104). Intramuscular injection of a eukaryotic expression plasmid expressing sPD-1 (pPD-1A) was shown to significantly inhibit the growth of H22 HCC cells in mice (62). Moreover, a test of local gene therapy showed that inoculation of a recombinant adeno-associated viral vector (rAAV)/sPD-1 construct at the site of H22 hepatoma cell inoculation inhibited tumor growth (59).

The combination of sPD-1 with other therapies demonstrated more robust antitumor effects than the other therapies alone. For example, local treatment of murine H22 HCC ectopic tumors by injection of naked plasmids expressing the co-stimulatory molecules 4-1BBL and sPD-1 eradicated a small number of pre-existing tumor cells in murine tumors and larger amounts of pre-existing tumor cells in approximately 60% of mice (105). An adenovirus expressing sPD-1-Ig markedly enhanced CD8^+^ T cell-mediated tumor rejection compared with adenovirus expressing herpes simplex virus thymidine kinase (HSVtk) alone (106). The fibronectin CH50-sPD-1 recombinant peptide was found to increase the cytolytic activity of cytotoxic T-lymphocytes and macrophages against tumors (60). Coadministration of sPD-1 with human papillomavirus-16 E7 DNA vaccine markedly improved E7-specific CD8^+^ T-cell responses and enhanced immunological cytotoxic killing effect on E7-expressing tumors (57). These findings demonstrate that sPD-1 plays a vaccine type-independent adjuvant role in suppressing tumor grown (57). In addition, sPD-1 enhanced the antitumor activity induced by other treatments, such as secondary lymphoid chemokines (SLC, CCL21) (107). Local gene transfer of IL-21 in combination with sPD-1 into murine H22 HCC tumors greatly facilitated the antitumor immune responses and inhibited tumor growth in mice (108). Similarly, co-delivery of miR-34a and sPD-1 by ultrasound complexed with microbubbles enhanced antitumor immunity and inhibited tumor tissue growth in mice bearing U14 cervical carcinomas (109). Pre-injection of a senescent tumor cell vaccine expressing sPD-1 (STCV/sPD-1) was found to protect mice from challenge with 4T1 murine breast cancer cells, delaying tumorigenesis and inhibiting early-stage tumor progression (110). Both *in vitro* and *in vivo* analyses demonstrated that high-affinity sPD-1 molecule blocked PD-L1- and PD-L2-mediated immune evasion and reduced tumor growth in immune-competent mouse models of ovarian cancer (111). Nanobubbles (NBs) containing sPD-1/dihydroporphyrin e6 (Ce6) were found to enhance tumor suppression by increasing tumor-targeted accumulation of Ce6 and sPD-1 and inducing ultrasound-targeted NB destruction (112). Transfection of tumor cells with sPD-1 delivered by NBs was found to downregulate PD-L1 expression, improving PD-1/PD-L1 signaling pathway-mediated T cell function (112). In addition, ICIs bound to Ce6 were shown to induce immunogenic cell death by translocating calreticulin to the cell surface, followed by synergistic enhancement of the antitumor immune response (112). A case-control analysis of patients with HBV-related HCC showed that median sPD-1 levels were significantly higher in men than in women patients (443.3 vs 307.3 pg/mL) (113). Moreover, high sPD-1 levels were associated elevated viral load, further increasing the risk of HCC in men (113). Taken together, these studies indicate that sPD-1 has potential clinical application in cancer treatment.

#### 5.2.2 Therapeutic Value of sPD-L1

The active sPD-L1 fragment generated by ADAM10 and ADAM17 cleavage of mPD-L1 was found to attenuate the killing of tumor cells by CD8^+^ T cells, revealing a novel mechanism of resistance to anti-PD-(L)1 antibodies (114). Two secreted PD-L1 splicing variants (PD-L1v242 and PD-L1v229) lacking the transmembrane domain were found to act as “decoys” of anti-PD-L1 antibodies, resulting in resistance to PD-L1 inhibitors (115). Anti-PD-1 antibodies, however, reversed the resistance induced by PD-L1 splicing variants (115). Small molecule inhibitors, such as BMS-1001 and BMS-1166, were found to impair the ability of sPD-L1 to inhibit T-lymphocyte activation (116). Because few studies have evaluated the biological activity of sPD-L1, no anticancer therapy directly targeting sPD-L1 has been tested in humans. Tumor-specific antigenic peptides presented by MHC molecules trigger TCR signaling and mediate T-cell activation. In addition, a deficiency in MHC molecules can result in sPD-L1 having subtle immunosuppressive effects (16). Targeting sPD-L1 alone may not effectively hamper tumor immune evasion (117). In-depth investigations of the functions of sPD-L1 and its use as a target for clinical anticancer therapy are urgently needed.

## 6 Exosomal PD-L1

In addition to PD-L1, which is free in solution, extracellular PD-L1 exists in another important form, exoPD-L1 (17, 118, 119). Exosomes are produced by double invagination of the plasma membrane and the formation of intracellular multivesicular bodies (MVBs) (120, 121). Following fusion of MVBs with the plasma membrane, these exosomes are secreted into the extracellular space and microenvironment by exocytosis (120, 121). Secreted exosomes transport their contents, including nucleic acids, lipids, and proteins, to target cells, thereby mediating intercellular signaling, drug resistance and immune regulation (122–126). Tumor cells can produce and secreted large amounts of exosomes with cancer-promoting contents (127, 128). Moreover, tumor-derived exosomes may act as regulatory elements that can reprogram the immune microenvironment (129, 130). Cancer-derived exosomes have been shown to present antigens to APCs to activate T-cell function and improve anti-tumor responses (130). ELISA and immunoelectron microscopy demonstrated that exoPD-L1 has the same membrane topology as mPD-L1, with many studies showing that tumor cell-derived exosomes carry bioactive PD-L1 and are able to bind to T cell surface PD-1 to deliver inhibitory signals (119). Exosomes also express other proteins that are crucial for T-cell signaling, such as MHC proteins, which interact with TCRs to deliver activation signals (17). Thus, exosomes co-expressing PD-L1 and MHC molecules may mediate more potent immunosuppressive effects than free sPD-L1 (131).

### 6.1 Immunosuppressive and Anti-Tumor Roles of ExoPD-L1

ExoPD-L1 inhibits immune cell-associated immune responses and promotes the growth of various tumor types. Replacing exogenously expressed PD-L1 on Raji B cells with exosomal PD-L1 from PC3 cells was shown to inhibit the activation of Jurkat T cells and reduce IL-2 secretion (118). Similarly, tumor-derived exoPD-L1 was found to inhibit T-cell activation in draining lymph nodes *in vivo* and the introduction of exogenous exoPD-L1 promoted tumor growth (118). *In vitro* co-culture of PD-L1^high^ exosomes with T cells effectively inhibited T cell activation and reduced CD69 expression on CD8^+^ T cells (132). ExoPD-L1 in the supernatant of murine or human HNSCC cell lines was found to reduce the infiltration of CD4^+^ and CD8^+^ T cells into tumor sites (133). In addition, exoPD-L1 levels correlated with disease progression in HNSCC patients (133). ExoPD-L1 derived from LLC Lewis lung or 4T1 breast cancer cells inhibited the differentiation of bone marrow precursor cells to DCs and their maturation, decreasing CD4^+^ IFN-γ^+^ Th1 differentiation while increasing the percentage of Tregs (134). Breast cancer-derived exosomes were able to translocate functional PD-L1, not only from PD-L1-positive to PD-L1-negative breast cancer cells, but also to other cell types, including macrophages and DCs, to regulate immune surveillance (135). ExoPD-L1 was found to significantly inhibit CD3/CD28-induced ERK phosphorylation and NF-κB activation in T cells in a dose-dependent manner and to reduce the secretion of IL-2 and of granzyme B, a marker of killing activity (135). PD-L1-expressing extracellular vehicles (EVs) of glioblastoma (GBM) cells have been reported to show dose-dependent effects, with low doses of EVs resulting in an immunostimulatory phenotype and higher doses of EVs having immunosuppressive effects (136). Similarly, exoPD-L1 in NSCLC patients inhibited CD8^+^ T cell activity in a dose-dependent manner, inducing CD8^+^ T cell apoptosis and reducing IL-2 and IFN-γ production (137).

### 6.2 Predictive and Prognostic Significance of ExoPD-L1

ExoPD-L1 has been identified as a biomarker of disease status and the clinical effects of immunotherapy. High levels of exoPD-L1 have been reported to correlate with advanced tumor stage, larger tumor size (> 2.5 cm), lymph node metastasis and distant metastasis in patients with NSCLC (138). In addition, exoPD-L1 has been associated with poor prognosis in patients with pancreatic ductal adenocarcinoma (139). Metastatic melanoma patients with a > 2.43-fold change in exoPD-L1 levels during immunotherapy were found to be more likely to respond successfully to treatment (119). The increase in exoPD-L1 during anti-PD-1 treatment is thought to result from IFN-γ stimulation produced by reinvigorated CD8^+^ T cells, indicating that the change in tumor-derived exoPD-L1 is an adaptive response of tumor cells to T cell regeneration (140). A >100 pg/mL change in exoPD-L1 concentration in melanoma patients was found to have a 91% positive predictive value for disease progression (141). Detection of exoPD-L1 and N-cadherin in the sera of patients with osteosarcoma may predict the progression of pulmonary metastases (142). Significantly elevated plasma levels of PD-L1-positive EVs may be a biomarker predictive of survival outcomes in patients with DLBCL (143).

### 6.3 Therapeutic Value of ExoPD-L1

Evidence in tumor-bearing mice suggests that exosome elimination mitigates tumor burden and enhances the potency of anti-PD-1/PD-L1 antibodies, with exoPD-L1 elimination restoreing T-cell activation (144). In an established xenograft mouse model of PD-L1 knockdown in the TRAMP-C2 cell line, the percentage of CD8^+^ T cells in draining lymph nodes was upregulated and the proportions of cells expressing granzyme B and Ki67 were increased, while the percentage of cells expressing the exhaustion marker Tim3 was downregulated (118). ExoPD-L1 deficiency not only inhibited local tumor growth but also blocked the ability of wild-type tumor cells to attack the other flank, indicating a potent anti-tumor memory response (118). Treatment of 4T1 cells with the exosomal secretion inhibitor GW4869 or tetracycline-induced Rab27 knockdown significantly inhibited tumor growth and enhanced anti-PD-L1 efficacy (135). These findings suggest that binding of exoPD-L1 to anti-PD-L1 antibodies prevents these antibodies from sequestering PD-L1 on the surface of tumor cells (131, 144). In addition, exosome removal from the circulation by hemofiltration may have anti-tumor effects (131). IFN-γ increases the amount of PD-L1 on exosomes released from metastatic melanoma, thereby inhibiting the function of CD8^+^ T cells, whereas anti-PD-1 antibodies reverse these effects and halt tumor progression (119). Similarly, anti-PD-1 antibodies were shown to significantly reverse the exosome-mediated blockade of T-cell activation in glioblastoma (145), suggesting that these anti-PD-1 antibodies compete with exoPD-L1 to bind to PD-1 on T cells (131). These findings suggest that exosome elimination may be a viable and necessary concomitant therapy for anti-PD-1/PD-L1 antibody potentiation.

## 7 Conclusion and Perspective

The PD-1/PD-L1 pathway is critical for inducing tumor escape from the immune system. Immunotherapy targeting PD-1/PD-L1 has revolutionized the paradigm of cancer therapy. Biomarkers predicting the efficacy of immunotherapy have also been extensively investigated (146–148). sPD-1/PD-L1 and exoPD-L1 molecules have been detected in the blood of cancer patients. sPD-1/PD-L1 are generated by proteolytic cleavage of membrane-bound molecules or by selective splicing during gene transcription. These molecules may be important components of immune regulation, although their exact biochemical properties and functions have not yet been determined. Several methods have been developed to assess these molecules. Current investigations have focused mainly on the use of these biomarkers to assess patient prognosis and to predict the efficacy of treatment (149, 150). Accumulated data indicate that these molecules may correlate with disease progression and patient survival. Changes in their levels after certain treatments, including surgery, radiotherapy, targeted therapy, and immunotherapy, suggest their potential as biomarkers for predicting treatment efficacy. In addition, survival may correlate with resistance to PD-(L)1 inhibitors. The characteristics of sPD-1 suggest that it may attenuate immunosuppression by blocking ligands. Lower sPD-1 levels have fewer side effects than monoclonal antibodies while exerting strong therapeutic effects. *In vivo* and *in vitro* studies have indicated that delivery of sPD-1 into the tumor microenvironment induces antitumor immunity. In addition, combination therapeutic strategies have shown synergistic effects. Similar to mPD-L1, sPD-L1 and exoPD-L1 bind to mPD-1 to exert immunosuppressive effects, suggesting that these reactions may be targets of anticancer treatment. Few studies to date have evaluated the potential of targeting sPD-1/PD-L1 and exoPD-L1 in cancer therapy. Results to date seem promising, suggesting new clinical antitumor strategies.

PD-1/PD-L1 blockade alone, however, is likely insufficient to obtain optimal results in a large proportion of patients. In addition to PD-1/PD-L1, other stimulatory and inhibitory molecules are involved in immune regulation. The various forms of these molecules, including soluble, exosome-associated, and membrane-associated molecules, play roles in the tumor microenvironment (27). For example, sCTLA-4 binds to the co-stimulatory ligand B7 on APCs and prevents B7 from binding to CD28 on T cells, thereby inhibiting T cell function (151). sCD86 is thought to bind to CTLA-4 and deliver negative signals to T lymphocytes (152). sB7-H3 not only significantly inhibits T cell proliferation but also activates NF-κB signaling by upregulating TLR4 expression, which induces IL-8 and VEGF expression, thereby promoting neovascularization and cancer cell invasion and metastasis (153). In addition, tumor-secreted sCD137 inhibits T-lymphocyte activation by blocking the interaction of CD137L with CD137 on T-lymphocytes (154). Additional studies of inhibitory and stimulatory molecules in cancer are necessary, including studies on the various forms of these molecules, their complex interactions, and their changes in concentration during tumor progression.

## Author Contributions

MN and YL drafted the manuscript and prepared the figure and tables. MY helped in revising it critically for important intellectual content. DJ and KW designed this review and revised the manuscript. All authors contributed to the article and approved the submitted version.

## Funding

This work was supported by the National Natural Science Foundation of China (No.81874120, 82073370).

## Conflict of Interest

The authors declare that the research was conducted in the absence of any commercial or financial relationships that could be construed as a potential conflict of interest.

## Publisher’s Note

All claims expressed in this article are solely those of the authors and do not necessarily represent those of their affiliated organizations, or those of the publisher, the editors and the reviewers. Any product that may be evaluated in this article, or claim that may be made by its manufacturer, is not guaranteed or endorsed by the publisher.
